# Amlodipine improves symmetric dimethylarginine in dogs with chronic kidney disease

**DOI:** 10.3389/fvets.2025.1570349

**Published:** 2025-04-30

**Authors:** Shohei Morita, Youhei Mochizuki, Takahiro Kondo, Yasuyoshi Matsuda, Takahiro Ohmori, Aritada Yoshimura, Ryuji Fukushima

**Affiliations:** ^1^Koganei Animal Medical Emergency Center, Tokyo University of Agriculture and Technology, Tokyo, Japan; ^2^Faculty of Veterinary Medicine, Okayama University of Science, Okayama, Japan; ^3^Animal Medical Center, Tokyo University of Agriculture and Technology, Tokyo, Japan

**Keywords:** amlodipine, blood pressure, chronic kidney disease, dog, hypertension, SDMA

## Abstract

**Introduction:**

In canines, chronic kidney disease (CKD) is frequently associated with high blood pressure. Amlodipine is used to treat hypertension in dogs, and we anticipated that amlodipine administration might improve renal function in dogs. However, the effect of amlodipine on canine renal function is unknown. Therefore, this study evaluated changes in symmetric dimethylarginine (SDMA) levels before and after amlodipine administration in pet dogs that had been diagnosed with CKD based on persistently elevated SDMA levels and were being treated with amlodipine alone for any reason. We also conducted a comparative investigation of whether there were any differences in SDMA changes depending on whether these dogs with CKD had hypertension.

**Methods:**

This study employed a retrospective design. The study subjects were pet dogs that exhibited persistently elevated SDMA (≥14 μg/dL), were diagnosed with CKD, and were treated with amlodipine. Profile data such as breed, sex, and age, as well as data on blood chemistry tests, blood pressure, heart rate, and echocardiograms before and after amlodipine administration, were collected. Forty-five dogs were included in the study, of which 20 were hypertensive (HT: systolic arterial pressure ≥160 mmHg) and 25 were non-hypertensive (Non-HT: systolic arterial pressure <160 mmHg).

**Results:**

Mean SDMA was significantly lower after drug administration compared with before administration in both the HT and Non-HT groups. Moreover, we found that cardiac output (CO) increased in all dogs with CKD treated with amlodipine. Blood pressure measurements showed that the blood pressure decreased in both the HT and Non-HT groups.

**Discussion:**

It is believed that the increase in CO due to amlodipine administration increases glomerular filtration rate, which may have led to a decrease in SDMA levels. Based on the rate of decrease in systolic arterial pressure, we considered that amlodipine might decrease blood pressure by a greater amount in patients with higher levels of hypertension. In this study, we showed that amlodipine administration improved SDMA in dogs with CKD regardless of whether they were hypertension. We also showed that amlodipine could be safely used to treat normotensive dogs.

## Introduction

Chronic kidney disease (CKD) is usually defined as a long-term abnormality in the structure or function of one or both kidneys that is present for more than 3 months (1). Canine CKD is characterized by the progressive decline of kidney function and has an overall reported prevalence of 0.05–3.74% (2, 3).

The International Renal Interest Society (IRIS) guidelines include symmetric dimethylarginine (SDMA) as a diagnostic indicator for canine CKD (4). Specifically, persistently elevated SDMA is one of the diagnostic criteria for distinguishing Stage 1 CKD from early Stage 2 (4). SDMA is significantly correlated with the glomerular filtration rate (GFR), and its level is believed to rise once approximately 40% of renal function has been lost (5). In human medicine, the treatment of patients with end-stage renal failure undergoing hemodialysis with the dihydropyridine calcium channel blocker (CCB) amlodipine reportedly reduces SDMA levels (6). Amlodipine administration has also been shown to increase GFR and renal plasma flow in kidney transplant patients (7).

In canines, CKD is frequently associated with high blood pressure, although it is difficult to determine whether this is a cause or effect (8). Amlodipine is used to treat hypertension in dogs, and the American College of Veterinary Internal Medicine (ACVIM) guidelines consider it the second-line drug (8). Based on these findings, we anticipated that amlodipine administration might improve renal function in dogs. Additionally, we thought that it may yield favorable outcomes in renal function improvement regardless of the presence or absence of hypertension. However, the effect of amlodipine on canine renal function is unknown.

Therefore, in this study, we evaluated changes in SDMA levels before and after amlodipine administration in pet dogs that had been diagnosed with CKD based on persistently elevated SDMA levels and were being treated with amlodipine alone for any reason. We also conducted a comparative investigation of whether there were any differences in SDMA changes depending on whether these dogs with CKD had hypertension.

## Materials and methods

### Study subjects

**Figure 1:**
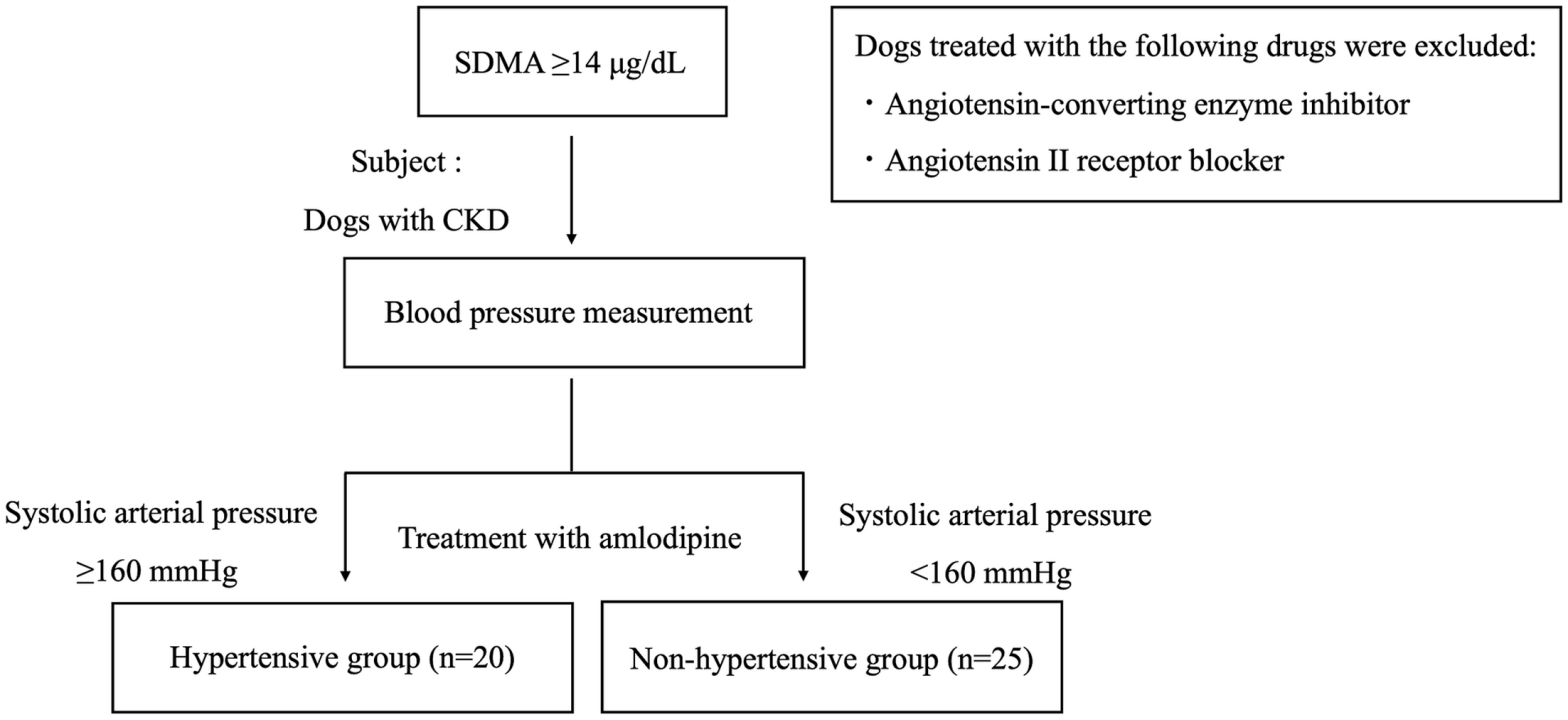
Flowchart of study subjects. The study involved dogs with chronic kidney disease treated with amlodipine. The subjects were also divided into two groups: a hypertensive group and a non-hypertensive group. CKD, chronic kidney disease; SDMA, symmetric dimethylarginine.

The study subjects were pet dogs brought by their owners to the Tokyo University of Agriculture and Technology Animal Medical Center or Koganei Animal Medical Emergency Center between November 2017 and December 2024 that exhibited persistently elevated SDMA (≥14 μg/dL), were diagnosed with CKD, and were treated with amlodipine (Figure 1). Dogs that had been administered a cardiovascular drug other than amlodipine before the start of amlodipine administration were included in the study. Dogs receiving treatment with angiotensin-converting enzyme inhibitors or angiotensin II receptor blockers were excluded. This study employed a retrospective design.

### Study parameters

We collected data on parameters including breed, sex, age (at the start of drug administration), weight, five-level body condition score (BCS) (9), blood biochemistry tests, blood pressure, rate of decrease (%) in systolic arterial pressure (SAP) after starting amlodipine [calculated as pre-administration SAP/(pre-administration SAP – post-administration SAP) × 100], heart rate, and echocardiography results. We also investigated the underlying disease, drug dose, concomitant drugs, side effects, as well as reasons for administering amlodipine to the study animals.

The blood biochemistry test parameters studied were blood urea nitrogen (BUN), serum creatinine (CRE), and SDMA levels before and after the start of drug administration. The measurement of SDMA levels was outsourced to IDEXX Laboratories (Enzyme-Linked Immunosorbent Assay). BUN and CRE were measured using DRI-CHEM (NX700V, FUJIFILM Corporation, Tokyo, Japan).

The echocardiographic parameters investigated were left ventricular internal diameter end-diastole (LVIDd), left ventricular internal diameter end-systole (LVIDs), fractional shortening (FS), left atrial-to-aortic root ratio (LA/Ao), stroke volume (SV), and cardiac output (CO).

End-diastolic volume (EDV) was calculated according to the following formula: EDV (mL) = 7 (LVIDd)^3^/(2.4 + LVIDd) (10). End-systolic volume (ESV) was calculated according to the following formula: ESV (mL) = 7 (LVIDs)^3^/(2.4 + LVIDs) (10). Systemic vascular resistance (SVR) was calculated according to the following formula, with central venous pressure (CVP) assumed to be 10 mmHg (11).

$SVR\;\left(\mathrm{dyne}\times \sec\times {\mathrm{cm}}^{-5}\right)=\left(\begin{array}{l} \mathrm{mean blood}\\ \mathrm{pressure}–CVP \end{array}\right)/CO\times 79.92$

Following the ACVIM guidelines, multiple blood pressure measurements were taken under resting conditions. Dogs with a SAP of ≥160 mmHg were classified as the hypertensive (HT) group (8). Blood pressure was re-evaluated 14–28 days later, leading to a final classification (Figure 1). Dogs with a SAP of <160 mmHg were classified as the non-hypertensive (Non-HT) group (Figure 1). CKD was classified according to the IRIS staging system (4). SDMA, BUN, and CRE were measured twice or more with an interval of at least 14 days from the first measurement. The presence or absence of CKD was determined based on the results. Additionally, when taking measurements on the same individual, care was taken to standardize the time of blood collection and meals.

### Designation of endpoints

The primary endpoint was renal failure-associated mortality. Renal failure-associated mortality was defined as diseases and related health problems included in codes N17–N19 and I12 of the International Classification of Diseases (ICD-10) (12).

The secondary endpoint was death from any cause, including renal failure-associated mortality, in animals that discontinued administration during the study and in those in which administration was ongoing but was censored. Survival time was defined as the number of days from the start of amlodipine administration until the endpoint was reached or censoring occurred.

### Statistical analysis

All measurements are expressed as the mean ± standard deviation. The time from amlodipine administration to the subsequent evaluation of each parameter and the survival times are expressed as the median and interquartile range (IQR). To confirm whether the measured values had a normal distribution, normal probability plotting was conducted, and a Kolmogorov–Smirnov test was used to test the normality of distribution. A paired *t*-test or Wilcoxon signed-rank test was then used to compare the pre-administration and post-administration values of each parameter in the same individual. Intergroup comparisons were conducted using an unpaired t-test or Mann–Whitney U test. For the IRIS stage, underlying disease, and side effects, intergroup comparisons were conducted by using a 𝜒^2^ test. Survival time analysis was conducted using univariate Cox proportional hazard analysis for the primary and secondary endpoints was used to evaluate the association between each variable and the time to reach the endpoint, and calculate the hazard ratio (HR) and 95% confidence interval (95%CI). Eight variables were designated: age (at renal failure diagnosis), weight, BCS, amlodipine dose, IRIS stage, SAP before and after the start of amlodipine administration, and complications of heart disease. Variables with a *p*-value of < 0.1 in univariate analysis were included in the multivariate Cox proportional hazard analysis. Multivariate Cox proportional hazard analysis was conducted by backward elimination, with all the remaining variables with a *p*-value of < 0.1 included in the final model. The HRs and 95%CIs for the variables remaining in the final model were calculated. A log-rank test was used to compare the survival rates of the HT and Non-HT groups, and Kaplan–Meier survival curves were prepared. All statistical analyses were conducted using computer statistical analysis software (SPSS Statistics version 25, Japan IBM, Tokyo, Japan), with *p* < 0.05 regarded as statistically significant in all cases.

## Results

### Overview of study subjects

Forty-five dogs were included in the study, of which 20 were HT and 25 were Non-HT. By breed, the HT group comprised five toy poodles, three miniature schnauzers, two mixed breeds, two chihuahuas, two Pembroke Welsh corgis, and six other breeds, while the Non-HT group comprised five toy poodles, four chihuahuas, three shih tzus, three miniature schnauzers, two mixed-breeds, and eight other breeds (Table 1).

**Table 1:** Composition of dog breeds in this study.

Breed	Group	
	HT	Non-HT
Toy poodle	5	5
Miniature schnauzer	3	3
Mixed breeds	2	2
Welsh corgi	2	0
Chihuahua	2	4
Maltese	1	0
Jack Russell terrier	1	1
Miniature Dachshund	1	0
Pekinese	1	0
Yorkshire terrier	1	1
Bichon Frise	1	0
Shih Tzu	0	3
Boston terrier	0	1
Shetland Sheepdog	0	1
Shiba Inu	0	1
Wire fox terrier	0	1
Pomeranian	0	1
Labrador retriever	0	1
Total	20	25

HT, hypertensive; Non-HT, non-hypertensive.

By sex, the HT group comprised two entire females, eight neutered males, and ten spayed females, whereas the Non-HT group comprised one entire male, two entire females, ten neutered males, and 12 spayed females (Table 2). The mean age at the start of amlodipine administration was 12 ± 3 years in the HT group and 12 ± 1 year in the Non-HT group (Table 2). The mean weight was 5.9 ± 2.8 kg in the HT group and 6.2 ± 3.8 kg in the Non-HT group (Table 2). In terms of the five-level BCS score, the HT group included one dog scoring 2/5, 12 scoring 3/5, and seven scoring 4/5, while the Non-HT group included one dog scoring 1/5, four scoring 2/5, 11 scoring 3/5, and nine scoring 4/5 (Table 2).

**Table 2:** Overview of the target group in this study.

Variable	Group	
	HT	Non-HT
Number of dogs	20	25
Sex (*n*)		
Male	0	1
Neutered male	8	10
Female	2	2
Spayed female	10	12
Age (years)	12 ± 3	12 ± 1
Weight (kg)	5.9 ± 2.8	6.2 ± 3.8
BCS (*n*)		
Scoring 1/5	0	1
Scoring 2/5	1	4
Scoring 3/5	12	11
Scoring 4/5	7	9
Scoring 5/5	0	0
Amlodipne dose (mg/kg/day)	0.27 ± 0.12	0.22 ± 0.09
IRIS staging system (*n*)		
Stage 1	13	20
Stage 2	7	4
Stage 3	0	1
Stage 4	0	0

Date are reported as mean ± SD. HT, hypertensive; Non-HT, non-hypertensive; IRIS, International Renal Interest Society.

In this study, 17 dogs in the HT group and 23 dogs in the Non-HT group had other diseases in addition to CKD. In the HT group, these diseases comprised seven cases of mitral valve insufficiency, five of chronic pancreatitis, four of ureteral calculus, three of tricuspid valve insufficiency, two of transitional cell carcinoma, two of renal calculus, and one each of adrenal tumor, hypothyroidism, trichoblastoma of the auditory canal, gallbladder mucocele, bladder calculus, pulmonary hypertension, and gallstone (Table 3). In the Non-HT group, the diseases comprised 17 cases of mitral valve insufficiency, five of chronic pancreatitis, four of tricuspid valve insufficiency, three of ureteral calculus, three of renal calculus, two of protein-losing enteropathy, two of aortic regurgitation, and one each of tracheal collapse, hyperadrenocorticism, biliary sludge, pulmonary regurgitation, diabetes, gallstone, bladder calculus, dilated cardiomyopathy, hypothyroidism, mammary tumor, and vestibular disease (Table 3). Two or more diseases were present in nine dogs in the HT group and 16 in the Non-HT group. The rate of mitral valve insufficiency was significantly higher in the Non-HT group than in the HT group (*p* = 0.03).

**Table 3:** Underlying diseases other than chronic kidney disease.

Disease	Group	
	HT	Non-HT
Mitral valve insufficiency	7	17
Chronic pancreatitis	5	5
Ureteral calculus	4	3
Tricuspid valve insufficiency	3	4
Transitional cell carcinoma	2	0
Renal calculus	2	3
Adrenal tumor	1	0
Hypothyroidism	1	1
Trichoblastoma of the auditory canal	1	0
Gallbladder mucocele	1	0
Bladder calculus	1	1
Pulmonary hypertension	1	0
Gallstone	1	1
Protein-losing enteropathy	0	2
Aortic regurgitation	0	2
Tracheal collapse	0	1
Hyperadrenocorticism	0	1
Biliary sludge	0	1
Pulmonary regurgitation	0	1
Diabetes	0	1
Dilated cardiomyopathy	0	1
Mammary tumor	0	1
Vestibular disease	0	1
Total	30	47

*There were 9 cases in the hypertension group and 16 cases in the non-hypertension group who had two or more diseases. HT, hypertensive; Non-HT, non-hypertensive.

The median time between evaluation of each parameter before and after the start of amlodipine administration was 28 days (IQR, 14–35 days) in the HT group and 28 days (IQR, 17–39 days) in the Non-HT group. There was no significant difference in the administration time between the groups.

The mean amlodipine dose was 0.27 ± 0.12 mg/kg/day in the HT group and 0.22 ± 0.09 mg/kg/day in the Non-HT group (Table 2). The dose was significantly higher in the HT group than in the Non-HT group (*p* = 0.02).

In this study, pimobendan was used as a cardiovascular-related drug in 13 dogs before amlodipine administration. These dogs had mitral valve insufficiency or other cardiovascular diseases. Other drugs used included trepibutone in 11 cases, ursodeoxycholic acid in nine, camostat mesilate in eight, and others including pancrelipase, levothyroxine sodium, and medicinal activated charcoal. There was no change in the dosage or administration of any of these drugs after the start of amlodipine administration, and no new medication was added. There was no significant difference in pimobendan use between the HT and Non-HT groups.

The only side effect observed in 45 dogs treated with amlodipine was a single case of gingival hyperplasia.

Reasons for administering amlodipine included the following: four cases in which gastrointestinal symptoms developed following the administration of an ACE inhibitor (ACEI), two cases in which using an ACEI would worsen a condition, such as during ongoing treatment for Addison’s disease or hyperadrenocorticism, nine cases in which ACEI administration did not result in blood pressure changes; six cases in which renal function markers increased after ACEI administration and 24 cases of mitral regurgitation where a strong left atrial pressure-reducing effect of amlodipine was expected.

### Blood biochemistry tests

**Figure 2:**
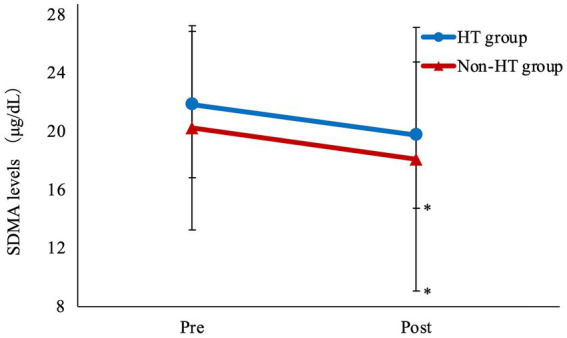
Amlodipine treatment improves SDMA levels Mean SDMA was significantly lower after drug administration compared with before administration in both the HT and Non-HT groups. HT, hypertensive; Non-HT, non- hypertensive; SDMA, symmetric dimethylarginine; Pre, before administration; Post, after administration. Pre vs. Post *p < 0.05.

Mean SDMA was significantly lower after drug administration compared with before administration in both the HT and Non-HT groups (Figure 2; Table 4). There was no significant difference in BUN or CRE levels after drug administration compared to before administration in either group. SDMA levels increased after drug administration in five dogs in the HT group and in three dogs in the Non-HT group. The HT group comprised 13 dogs that were at IRIS stage 1 and seven that were at stage 2, with no dogs at stage 3 or 4 (Table 2). The Non-HT group comprised 20 dogs at stage 1, four at stage 2, and one at stage 3, with no dogs at stage 4 (Table 2).

**Table 4:** Changes in blood biochemistry tests before and after amlodipine administration.

Variable	Group					
	HT			Non-HT		
	Pre	Post	*p* value	Pre	Post	*p* value
SDMA (μg/dL)	21.8 ± 7.7	19.8 ± 7.0	0.0349	20.2 ± 6.7	18.1 ± 9.0	0.0163
BUN (mg/dL)	35.9 ± 29.7	34.9 ± 18.9	0.9405	37.4 ± 19.9	41.6 ± 26.6	0.1500
CRE (mg/dL)	1.46 ± 0.68	1.42 ± 0.67	0.6979	1.31 ± 0.73	1.39 ± 0.82	0.2904

HT, hypertensive; Non-HT, non-hypertensive; SDMA, symmetrical dimethylarginine; BUN, plasma urea nitrogen; CRE, plasma creatinine; Pre, before administration; Post, after administration.

There was no significant difference in SDMA levels before and after amlodipine administration between the HT and Non-HT groups. The rate of decrease in SDMA levels after amlodipine administration was 8.0 ± 20.5% in the HT group and 12.8 ± 19.2% in the Non-HT group, which was not significantly different.

There was also no significant difference in the number of dogs at each IRIS stage between the two groups.

### Blood pressure measurements

In the HT group, SAP, mean arterial pressure (MAP), and diastolic arterial pressure (DAP) decreased significantly after drug administration compared to before administration (Table 5). In the Non-HT group, MAP and DAP decreased significantly after drug administration compared to before administration (Table 5).

**Table 5:** Changes in blood pressure levels and heart rate before and after amlodipine administration.

Variable	Group					
	HT			Non-HT		
	Pre	Post	*p* value	Pre	Post	*p* value
SAP (mmHg)	177 ± 13	148 ± 15	< 0.001	139 ± 10	135 ± 16	0.1243
MAP (mmHg)	134 ± 15	113 ± 10	< 0.001	105 ± 12	99 ± 14	0.0064
DAP (mmHg)	111 ± 18	94 ± 10	< 0.001	88 ± 14	82 ± 14	0.0096
Heart rate (bpm)	153 ± 40	166 ± 29	0.0553	132 ± 26	143 ± 23	0.0160

HT, hypertensive; Non-HT, non-hypertensive; SAP, systolic arterial pressure; MAP, mean arterial pressure; DAP, diastolic arterial pressure; Pre, before administration; Post, after administration.

**Figure 3:**
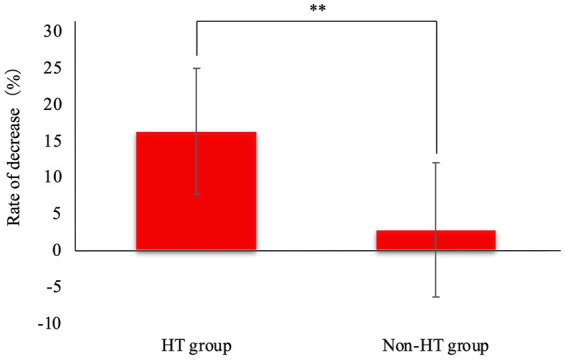
Rate of decrease in systolic arterial pressure after amlodipine administration. The rate of SAP decrease was significantly higher in the HT group than in the Non-HT group. HT, hypertensive; Non-HT, non- hypertensive; SAP, systolic arterial pressure. **p < 0.01.

The rate of SAP decrease was significantly higher in the HT group than in the Non-HT group (Figure 3). In the Non-HT group, SDMA decreased in 22/25 dogs, and the lowest SAP before amlodipine administration was 123 mmHg. SDMA increased in only three dogs, among which SAP before amlodipine administration was 125, 126, and 140 mmHg, respectively.

In the HT group, the mean heart rate was 153 ± 40 bpm before administration and 166 ± 29 bpm after administration, a change that was not significant (Table 5). However, in the Non-HT group, the mean heart rate was 132 ± 27 bpm before administration and increased significantly to 143 ± 25 bpm after administration (*p* = 0.02, Table 5).

### Echocardiography

In the HT group, CO and FS increased significantly, and LVIDd, LVIDs, EDV, ESV, and SVR decreased significantly after administration compared with before administration (Table 6). In the Non-HT group, SV and CO increased significantly, and LA/Ao and SVR decreased significantly after administration compared with before administration (Table 6). Overall, in dogs treated with amlodipine, SV and CO increased significantly, and LA/Ao, LVIDs, ESV, and SVR decreased significantly after administration compared with before administration (Table 6). The results and their significant differences are summarized in Table 6.

**Table 6:** Changes in echocardiography before and after amlodipine administration.

Variable	Group								
	HT			Non-HT			Total cases		
	Pre	Post	*p* value	Pre	Post	*p* value	Pre	Post	*p* value
LVIDd (mm)	22.0 ± 4.5	20.6 ± 4.1	0.0440	24.5 ± 6.8	24.9 ± 7.5	0.6926	23.3 ± 6.0	23.0 ± 6.7	0.1993
LVIDs (mm)	13.1 ± 3.2	11.0 ± 2.4	< 0.001	13.9 ± 5.7	13.5 ± 6.2	0.4470	13.5 ± 4.8	12.5 ± 5.1	0.0109
FS (%)	41.0 ± 7.0	46.0 ± 8.6	0.0304	44.5 ± 9.1	46.5 ± 11.5	0.3515	43.1 ± 8.5	46.1 ± 10.3	0.0590
EDV (mL)	17.4 ± 7.7	14.6 ± 6.9	0.0269	24.0 ± 19.2	25.4 ± 19.8	0.9515	21.1 ± 15.5	20.8 ± 16.4	0.2064
ESV (mL)	4.7 ± 2.8	2.9 ± 1.6	< 0.001	6.8 ± 11.8	6.7 ± 11.4	0.5230	5.9 ± 9.1	5.1 ± 8.8	0.0115
LA/Ao	1.04 ± 0.15	1.00 ± 0.08	0.2780	1.17 ± 0.28	1.08 ± 0.16	0.0097	1.12 ± 0.24	1.05 ± 0.14	0.0239
SV (mL)	6.60 ± 3.49	8.55 ± 5.59	0.1145	6.12 ± 4.63	7.60 ± 4.56	0.0140	6.32 ± 4.15	7.99 ± 4.97	0.0035
CO (L/min)	0.81 ± 0.27	1.24 ± 0.62	0.0042	0.73 ± 0.61	0.96 ± 0.51	0.0140	0.76 ± 0.49	1.08 ± 0.57	< 0.001
SVR (dyne × sec × cm-5)	13,658 ± 5,630	8,512 ± 4,671	< 0.001	14,551 ± 6,971	9,271 ± 4,791	< 0.001	14,180 ± 6,389	8,956 ± 4,698	< 0.001

LVIDd, left ventricular end-diastolic diameter; LVIDs, left ventricular end-systolic diameter; FS, left ventricular diameter fractional shortening; EDV, End-diastolic volume; ESV, End-systolic volume; LA/Ao, left atrial-aortic diameter ratio; SV, stroke volume; CO, cardiac output; SVR, systemic vascular resistance; Pre, before administration; Post, after administration.

### Endpoints

Of the 45 dogs included in this study, 10 had reached the primary endpoint at the end of the study. The secondary endpoint was reached in 44 dogs (ten cases of renal failure-related mortality, six of mortality unrelated to renal failure, two of discontinuation of medication, and 26 of continued administration). The causes of death other than renal failure-associated mortality included two cases of thromboembolism, one of cerebral infarction, one of transitional cell carcinoma, one of Sertoli cell tumor, and one of death from old age.

### Survival analysis

In univariate Cox proportional hazard analysis of the primary endpoint, the variables with *p* < 0.1 included amlodipine dose (HR 13.636, 95%CI 1.128–164.636, *p* = 0.034), IRIS stage (HR 3.907, 95%CI 1.692–9.026, *p* = 0.001), and SAP after amlodipine administration (HR 1.029, 95%CI 1.000–1.059, *p* = 0.051) (Table 7). Age (HR 1.182, 95%CI 0.961–1.454, *p* = 0.114), weight (HR 1.051, 95%CI 0.937–1.180, *p* = 0.392), BCS (HR 0.606, 95%CI 0.331–1.110, *p* = 0.104), SAP before amlodipine administration (HR 1.004, 95%CI 0.986–1.023, *p* = 0.640), and complications of heart disease (HR 0.491, 95%CI 0.184–1.307, *p* = 0.154) were not significant variables.

**Table 7:** Hazard ratio of reaching primary and secondary endpoints according to univariate Cox proportional hazards analysis.

Variable	Primary endpoint			Secondary endpoint		
	HR	95% CI	*p* value	HR	95% CI	*p* value
Age	1.182	0.961–1.454	0.114	1.221	1.012–1.474	0.026
Weight	1.051	0.937–1.180	0.392	1.057	0.959–1.166	0.264
BCS	0.606	0.331–1.110	0.104	0.771	0.414–1.222	0.276
IRIS stage	3.907	1.692–9.026	0.001	3.075	1.488–6.353	0.001
Amlodipine dose	13.636	1.128–164.636	0.034	6.507	0.606–69.924	0.117
SAP before amlodipine administration	1.004	0.986–1.023	0.640	1.006	0.990–1.023	0.466
SAP after amlodipine administration	1.029	1.000–1.059	0.051	1.013	0.989–1.038	0.282
Complications of heart disease	0.491	0.184–1.307	0.154	0.585	0.256–1.336	0.203

HR, hazard ratio; 95% CI, 95% confidence interval; BCS, body condition score; IRIS, international renal interest society; SAP, systolic arterial pressure.

In multivariate Cox proportional hazard analysis of the primary endpoint, including amlodipine dose, IRIS stage, and SAP after amlodipine administration, amlodipine dose (HR 34.725, 95%CI 1.326–909.611, *p* = 0.033) and IRIS stage (HR 6.776, 95%CI 2.406–19.081, *p* < 0.001) were significant variables (Table 8). SAP after amlodipine administration (HR 1.015, 95%CI 0.987–1.043, *p* = 0.291) was not significant.

**Table 8:** Hazard ratio of reaching primary and secondary endpoints according to multivariate Cox proportional hazards analysis.

Variable	Primary endpoint			Secondary endpoint		
	HR	95% CI	*p* value	HR	95% CI	*p* value
Age	–	–	–	1.187	0.969–1.454	0.099
IRIS stage	6.776	2.406–19.081	< 0.001	2.705	1.287–5.687	0.009
Amlodipine dose	34.725	1.326–909.611	0.033	–	–	–
SAP after amlodipine administration	1.015	0.987–1.043	0.291	–	–	–

HR, hazard ratio; 95% CI, 95% confidence interval; IRIS, international renal interest society; SAP, systolic arterial pressure.

In univariate Cox proportional hazard analysis of the secondary endpoint, the variables with *p* < 0.1 were age (HR 1.221, 95%CI 1.012–1.474, *p* = 0.026) and IRIS stage (HR 3.075, 95%CI 1.488–6.353, *p* = 0.001) (Table 7). Weight (HR 1.057, 95%CI 0.959–1.166, *p* = 0.264), BCS (HR 0.771, 95%CI 0.414–1.222, *p* = 0.276), amlodipine dose (HR 6.507, 95%CI 0.606–69.924, *p* = 0.117), SAP before amlodipine administration (HR 1.006, 95%CI 0.990–1.023, *p* = 0.466), SAP after administration (HR 1.013, 95%CI 0.989–1.038, *p* = 0.282), and complications of heart disease (HR 0.585, 95%CI 0.256–1.336, *p* = 0.203) were not significant variables.

Multivariate Cox proportional hazard analysis of the secondary endpoint, including age and IRIS stage, found that IRIS stage (HR 2.705, 95%CI 1.287–5.687, *p* = 0.009) was the only significant variable, whereas age (HR 1.187, 95%CI 0.969–1.454, *p* = 0.09) was not significant (Table 8).

**Figure 4:**
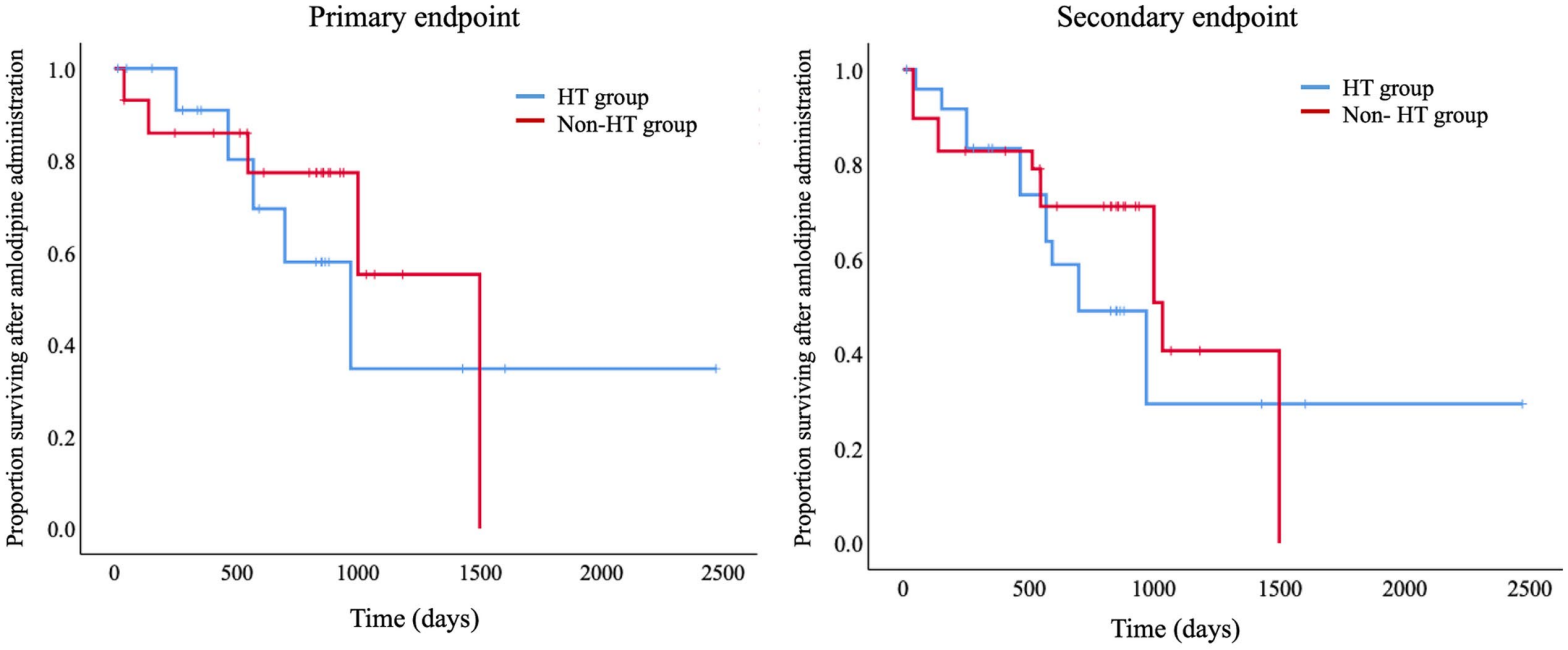
Kaplan–Meier curves for the primary and secondary endpoints. Log-Rank tests comparing survival times between the HT and Non-HT groups showed no significant differences between the two groups for either the primary or secondary endpoints. Primary endpoint, renal failure-associated mortality; Secondary endpoint, death from any cause, including renal failure-associated mortality; HT, hypertensive; Non-HT, non-hypertensive.

A comparison of the survival times for the HT and Non-HT groups using a log-rank test revealed no significant difference between the two groups with respect to either the primary or secondary endpoint (Figure 4). The overall median survival time to the primary endpoint was 559 days (IQR, 3076–903 days).

## Discussion

Suzuki et al. reported that when mitral valve insufficiency model dogs were treated with amlodipine, SVR and LA/Ao decreased significantly, and an actual decrease in left atrial pressure was observed (13). They concluded that this was due to the vasodilating action of amlodipine (which decreases the afterload) (13). In this study, similar to Suzuki et al., we found that SVR and LA/Ao decreased in the Non-HT group. These findings show that although myxomatous mitral valve disease (MMVD) and CKD differ in their pathophysiology, the use of amlodipine in dogs can reduce afterload and alleviate left atrial pressure.

An increase in FS is caused by decreased afterload, increased preload, increased systolic pressure, or a combination of these factors (14). In this study, a significant decrease in SVR was observed in the HT and Non-HT groups, as well as all animals administered amlodipine. In addition, a decrease in LVIDs and ESV was observed in the HT group, as well as all animals administered amlodipine. Based on these, it was determined that afterload decreased due to the vasodilating action of amlodipine. On the other hand, a significant decrease in LVIDd and EDV was observed only in the HT group, while no significant changes were observed in all animals administered amlodipine or the Non-HT group. This suggests that FS primarily increases due to reduced afterload or enhanced contractility, rather than preload reduction.

In this study, we found that CO increased in all dogs with CKD treated with amlodipine, regardless of hypertension status. SV also increased in dogs with CKD overall and in the Non-HT group. The heart rate increased significantly in the Non-HT group and tended to increase in the HT group (*p <* 0.06). Since CO is defined as SV × heart rate (14), the increase in CO was considered to be due to the synergistic effect of the increases in SV and heart rate caused by amlodipine administration. SDMA is inversely proportional to GFR. Thus, a decrease in SDMA signifies an increase or improvement in GFR. Because the blood volume that goes to the kidneys is generally approximately 20–25% of CO (15), increasing CO by means of amlodipine administration also increases the blood flow to the kidneys, and as GFR also increases, this is believed to have led to decreased SDMA levels. CRE levels increase when approximately 75% of renal function is lost (16). At IRIS stage 1, CRE level fluctuations are limited to within the reference values [4]. In this study, dogs at stage 1 in IRIS stage classification accounted for most of the subjects (73%), which might have led to no significant changes in CRE levels.

In this study, we found a few minor differences between the HT and Non-HT groups in terms of whether significant differences were present in several echocardiographic parameters. However, in both groups, changes in hemodynamics and cardiac morphology were generally caused by vasodilation. These differences between the two groups may have been further diminished by varying the amlodipine dose.

Reflex tachycardia is a biological reflex mechanism that excites the sympathetic nerves governing the heart and suppresses parasympathetic nerve excitation when blood pressure suddenly drops, increasing the heart rate and contractile force to increase cardiac output. In human medicine, reflex tachycardia is a general side effect of dihydropyridine L-type CCBs and is known to appear after their administration (17, 18). Among these drugs, amlodipine is known to have a low rate of reflex tachycardia (19). Nonetheless, a significant increase in the heart rate was evident in the Non-HT group in our study. A tendency for the heart rate to increase was also observed in the HT group, although this difference was not significant (*p =* 0.06). Sympathetic nerve activity is higher in human CKD patients (20). Therefore, we considered that the administration of amlodipine to clinical cases of canine CKD might affect the heart rate.

Blood pressure measurements showed that the blood pressure decreased in both the HT and Non-HT groups. Based on the rate of decrease in SAP, we considered that amlodipine might decrease blood pressure by a greater amount in patients with higher levels of hypertension. The benzodiazepine CCB, diltiazem, and the phenylalkylamine CCB, verapamil, block calcium channels via a use-dependent blocking modality (21). However, dihydropyridine CCBs generally lack this use-dependent blocking effect (22). Nonetheless, amlodipine is highly fat soluble, indicating that its dissociation from the channel is slow. This suggests that if the use-dependent block effect were somehow to be brought into use, the vasodilating effect might be more strongly exerted when the pre-administration blood pressure was higher, as in the present study. In this study, the Non-HT group exhibited an 88% improvement in SDMA; based on SAP values before amlodipine administration, it may also be possible to safely use amlodipine for Non-HT dogs if SAP is ≥125 mmHg.

The ACVIM guidelines state that amlodipine, the drug investigated in this study, is the second-line drug for the treatment of canine hypertension, with the first-line drug being an angiotensin-converting enzyme (ACE) inhibitor or another renin-angiotensin-aldosterone system (RAAS) inhibitor (8). In this study, amlodipine was administered instead of an ACEI to the study animals for various reasons. On the other hand, a study reported that the use of amlodipine alone did not lower mean blood pressure in healthy dogs (23). In the present study, however, the use of amlodipine alone resulted in a significant decrease in blood pressure in dogs with clinical hypertension. This may have been due to the difference between healthy and HT dogs or because the use-dependent block effect described above came into action. Park et al. reported that the administration of amlodipine produced no obvious side effects in 24 dogs with myxomatous mitral valve degeneration and signs of congestion (24). Geigy et al. reported that the administration of amlodipine did not cause hypotension or other side effects in 22 dogs with hypertension caused by acute kidney injury (25). The subjects in this study had a variety of diseases in addition to CKD, but we observed no obvious side effects of amlodipine administration, suggesting that it may be administered relatively safely even in non-hypertensive dogs. On the other hand, the safety of administering amlodipine to dogs has not yet been established, so the presence or absence of side effects must be carefully evaluated for each individual case.

Amlodipine is considered to be a very safe drug for the treatment of hypertension in dogs, with few side effects (8). Post-administration gingival hyperplasia is one such side effect, and Thomason et al. reported that its incidence in dogs treated with amlodipine for valvular disease was 8.5% (7/82) (26). In this study, the only side effect suspected to be associated with amlodipine administration was gingival hyperplasia in one dog (2.2%), a lower incidence than that reported by Thomason et al. This may be because the study population included a large number of dogs with conditions other than valvular diseases.

In the univariate Cox proportional hazard analysis, a higher amlodipine dose, lower SAP after amlodipine administration, and later IRIS stage were found to increase the risk of reaching the primary endpoint. Increasing age and later IRIS stage tended to increase the risk of reaching the secondary endpoint. In the multivariate Cox proportional hazard analysis, however, a later IRIS stage was the only factor that increased the risk of reaching either the primary or secondary endpoint. According to Rudinsky et al., survival time changes as the IRIS stage progresses (27). Therefore, as reported by Rudinsky et al., a later IRIS stage appears to be closely associated with the risk of death.

King et al. previously reported that there was no significant difference in the survival time of dogs with CKD treated with benazepril, an ACE inhibitor (median 305 days), and those given a placebo (median 287 days) (28). However, in our survival analysis, the overall median survival time at the primary endpoint was 559 days. Although the results of our study and those of ACE inhibitors cannot be compared directly, amlodipine treatment for dogs with CKD may potentially extend the survival time.

This study has several limitations. The first is its retrospective design; therefore, the time from amlodipine administration to evaluation varied for each dog. The results might have had less variation and could have been more accurate if we were able to stipulate the time from amlodipine administration to evaluation in advance. Second, we were unable to investigate proteinuria, which is believed to be a prognostic factor for both CKD and hypertension (8, 29). One reason ACEIs are considered the first-line treatment for hypertension in dogs, according to guidelines, is that hypertension is often accompanied by CKD, and ACEIs have been shown to reduce proteinuria, a prognostic factor for CKD (8). In this study, due to its retrospective nature, there were an insufficient number of cases in which urine protein levels were measured for statistical analysis. As a result, it was not possible to determine whether amlodipine treatment had a proteinuria-reducing effect or to assess the extent of such an effect. If we had investigated this, we might have ascertained the renoprotective effects of amlodipine in greater detail. Third, we were unable to investigate the individual elements of RAAS. Studies in healthy dogs have shown that RAAS is activated (leading to increased urinary aldosterone excretion) by the administration of amlodipine alone (23). Because RAAS overactivation can induce and increase the remodeling of renal and cardiac tissues and cause disorders, including high blood pressure and exacerbation of heart failure, further studies are required to confirm changes in RAAS in clinical cases.

In this study, we showed that amlodipine administration improved SDMA in dogs with CKD regardless of whether they were HT. We also showed that amlodipine could be safely used to treat normotensive dogs.

## Data Availability

The original contributions presented in the study are included in the article/supplementary material, further inquiries can be directed to the corresponding author.
